# Relationship of different intensities of physical activity and quality of life in postmenopausal women

**DOI:** 10.1186/s12955-020-01377-1

**Published:** 2020-05-06

**Authors:** Juliana Felipe, Juliana Viezel, Andréa Dias Reis, Emili Amice da Costa Barros, Thais Reis Silva de Paulo, Lucas Melo Neves, Ismael Forte Freitas Júnior

**Affiliations:** 1 Center of Studies and Laboratory of Evaluation and Prescription of Motor Activities (CELAPAM), Department of Physical Education, Sao Paulo State University (UNESP) / FCT, Rua Roberto Simonsen, 305, Presidente Prudente, SP 1960-900 Brazil; 2 Post Graduation Program in Motricity Science, São Paulo State University (UNESP), School Technology and Science, Presidente Prudente, SP Brazil; 3 Post Graduation in Physiotherapy, São Paulo State University (UNESP), School Technology and Science, Presidente Prudente, SP Brazil; 4 Department of Physical Education, Federal University of Amazonas (UFAM), Parintins, AM Brazil; 5 School of Physical Education, University of São Paulo (USP), São Paulo, SP Brazil

**Keywords:** Quality of life, Level of physical activity, Postmenopausal women

## Abstract

**Background:**

It is known that the elderly population remains most of the time in light activity. Physical activity plays a key role in the primary prevention of chronic diseases to mitigate various deleterious effects of aging and improve quality of life. The objective of the present study was to evaluate whether the time that postmenopausal women remain in light activities during the day are related to better quality of life and compare these results with the quality of life of those who remain longer in moderate intensity and vigorous activity.

**Methods:**

This is a cross sectional study there were evaluated 102 women, aged 50 to 79 years, all postmenopausal. Physical activity was measured by triaxial accelerometers. The quality of life was assessed using a Brazilian validated version of the SF-36 questionnaire. The sample was divided in three groups (G1, G2 and G3) according to tercile of time spent per week on light, moderate and moderate+vigorous physical activity. The comparisons between groups were made by ANOVA One Way, and the relationship between variables were made through the Spearman’s correlation coefficient, and the significance was set at 5%.

**Results:**

We found that the amount of time of light physical activity shows a higher correlation values compared to the moderate and moderate+vigorous physical activity (*p* < 0,05) and presented significant correlation in all domains of quality of life. Vigorous physical activity did not presented significant correlation in all domains of quality of life.

**Conclusion:**

Our data suggests that light intensity physical activity presented influence on the quality of life of postmenopausal women.

**Trial registration:**

(NCT02804308). Registered on 17 june 2016 (retrospectively registred).

## Introduction

Menopause is the period marked by significant changes in women, including changes in body composition, with total body mass gain that occurs as a result of increased total and central body fat, but also a decrease in lean body mass [1, 2]. Other behavioral character changes can affect directly personal and social, directly influencing the quality of life [3].

These changes cause a decrease in level of habitual physical activity and influence directly on the negative symptoms of menopause and aging in woman [4]. The American College of Sports Medicine recommends that adults perform 150 min/week of moderate physical activity for maintaining health [5], since studies report that compliance with such a recommendation will also help in maintaining body composition and will consequently result in a better quality of life [6].

Sedentary lifestyle is a condition that affects a large part of the population of postmenopausal women, further aggravating the consequences inherent to aging [4]. On the other hand, physically active people have longer longevity and lower morbidity and mortality rates.

Postmenopausal women with sedentary behavior have decreased resistance, strength, and lower limb muscle velocity [7]. In addition, muscle weakness and low cardiorespiratory capacity are responsible for a slower gait [8] and less safety. It is also a factor that decreases walking frequency in activities of daily living, generating a longer time of inactivity [9].

In this context, the symptoms and diseases related to human aging and menopause can be minimized through the practice of physical exercises, in the various intensities (mild, moderate and intense), types (aerobic, anaerobic and combined), and adoption of other healthy living habits [10].

However, it is known that the elderly population remains most of the time in mild activity and that these activities, such as walking, are more beneficial to maintaining quality of life than performing activities of moderate or vigorous intensity this population. Therefore, physical activity plays a fundamental role in the primary prevention of chronic diseases to attenuate several deleterious effects of aging [9].

In addition, mild physical activity seems to improve the psychic sphere, consequently favors a better quality of life [11]. However, little is known about the relationship between mild physical activity and the domains of quality of life in postmenopausal women, as well as the benefits provided in the different intensities (mild, moderate and moderate + vigorous) of physical activity to this population.

Therefore, the objective of the present study was to evaluate whether the time that postmenopausal women remain in mild activities during the day is related to the better quality of life and to compare these results with the quality of life of those who remain longer in moderate activity of intensity and vigorous.

## Methods

### Sample

This cross sectional study evaluated 102 women, aged between 50 and 79 years, all postmenopausal, who participated in the exercise intervention program developed between 2014 to 2016, at the São Paulo State University (UNESP), campus of Presidente Prudente, São Paulo. The data refers to the baseline moment of two clinical trials developed during 2014 to 2016, which had the objective investigating the effects of functional and combined training on body composition, metabolic profile, physical fitness, physical activity and QoL of postmenopausal women [12, 13].

In order to be included in these clinical trials, participants had to meet the following criteria: 1) menopausal period (no menstrual cycle for 12 consecutive months or more); 2) age between 50 and 79 years old at the day of the evaluation; 3) do not present physical limitations or any health problem that prevents to perform all evaluations or daily PA; 4) sign the consent form and formal clarification for participation in the study. The sample size calculator was used for sample inference, using a confidence level of 95%, alpha error 5%. A population proportion 10% and population size 207.610 (populational census of Presidente Prudente in 2010). Yielding the results for a new study of 84 woman, with 1:1 division of groups (G1 *n* = 28, G2 n = 28, G3 n = 28).

The invitation to participate in both studies was made through data base of regional cancer hospital, groups of cancer patients, and local media (radio, television and newspapers). The procedures used in this research attended the Criteria of Ethics in Research with Human Beings according to resolution n. 196/96 of the National Health Council - Brasília – DF and all subjects included in this study signed the Informed Consent form. The present study was approved by the Ethics and Research Committee of São Paulo State University (UNESP) (numbers 11,547,013.2.0000.5402 and 6,727,715.1.0000.5402/2015), and registered on ClinicalTrials.gov (NCT02804308) and on Brazilian registration clinical trial (RBR-85vmkz).

### Data collection

#### Physical activity

The habitual physical activity (PA) was measured by triaxial accelerometers motion sensor Actigraph, GT3X model (Actigraph LLC, Pensacola, FL). The devices are light, small and designed to be positioned in the participant to record the movements in the three orthogonal planes. The equipment measures and records acceleration variations whose magnitudes cover approximately 0.05 to 2.5 g (g = 9.8 m/s^2^) within a frequency range of 0.25 to 2.5 Hz. Each data sample (counts) was summed over a particular time interval, called the epoch, of 60 s. This period of 60 s was chosen because it is the most related to the pattern of activity of low intensity and long duration [9].

The volunteers were instructed to wear the device at the waist and remain with the equipment for, at least, seven full days (five weekdays and two weekend days). Participants were advised to take care of the equipment, which should be kept during daytime in the waking hours, and only removed when performing an activity with water (personal hygiene or water activities) and while sleeping.

For analysis of accelerometer data was used the specific software, ActiLife6 - Data Analysis Software by Actigraph. It was included in the database only full days of monitoring with at least ten hours of use and participants with at least five days of monitoring.

The raw accelerometer data were translated in minutes of physical activity and its intensity was analyzed according to recommendations [10] for accelerometer triaxial. Mild physical activity (< 3.00 METs) was defined as less than 1951 counts per minute; moderate physical activity between 1952 and 5724 (3.00–5.99 METs); vigorous physical activity between the range of 5725 to 9498 (6.00–8.99 METs); and very vigorous physical activity comprised in values greater than 9499 counts per minute (9 METs).

The habitual physical activity were expressed in minutes per week of mild, moderate, moderate+vigorous physical activity (MVPA), and counts per minute (the sum of counts of the three accelerometer axis divided by the time used).

#### Quality of life

Health-related quality of life was assessed using a Brazilian validated version of the SF-36 questionnaire [11]. This instrument contains 36 items divided into eight dimensions (functional capacity, pain, physical aspects, emotional aspects, social aspects, mental health, vitality and general health status). For each dimension, SF-36 items were coded, grouped and transformed into a scale of 0 (worst health) to 100 (best health status).

### Statistical analysis

To verify the normality of the data the Kolmogorov-Smirnov test was used. Data are presented as descriptive statistics, expressed as means and standard deviations. The sample was divided in three groups (G1, G2 and G3) according to tercile of time spend per week in mild, moderate and MVPA. The comparisons between groups were made using the one-way ANOVA test. The Pearson correlation coefficient was used to verify the association between the time of different physical activity intensities and Qol. All analyzes were performed using the statistical package SPSS version 21.0, with a significance level set at 5%.

## Results

**Fig. 1:**
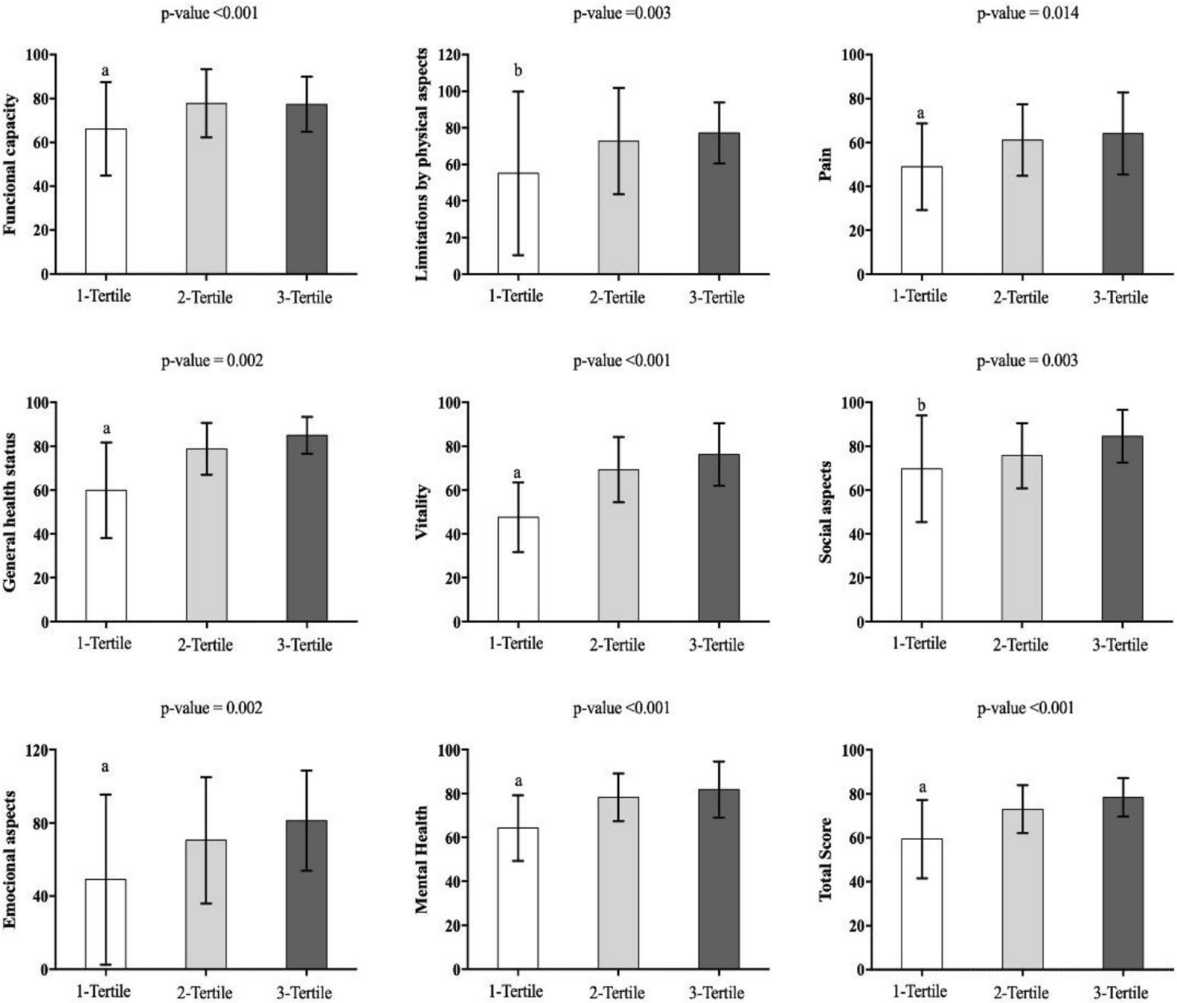
Quality of life women postmenopausal women allocated according to light physical activity per week (n = 102)

Study participants had a mean age of 62.1 ± 7.8 years and the proportion of mild to moderate activity was 23.2 ± 33.5 min/day per week (minimum: 3.8; maximum: 245.3). That is, on average, for each minute in moderate physical activity per day, the participants performed 23 min in mild physical activity. The mean time spent in mild activity in the three groups studied presented a statistical difference (f = 245.027, p = < 0.001). All the domains of QoL presented a difference, emphasizing G1 that presented higher values ​​in almost all domains (functional capacity (f = 0.172, *p* = 0.003), pain (f = 6.520, *p* = 0.002), general health status (f = 25.290, *p* < 0.001), vitality (F = 33.496, *p* < 0.001), emotional aspects (f = 6.721, p = 0.002), mental health (f = 17.348, *p* < 0.001) and overall score (f = 23,353, *p* < 0.001) when compared to G2 and G3. Only in the domains limitation by physical aspects (f = 4446, p = 0,014) and social aspects (f = 6.015, p = 0.003), G1 differs only from G3 (Fig. 1).

The time spent in moderate physical activity presented statistical difference (f = 209,867, *p* < 0.001) among the three groups. With regard to social and emotional domains of QoL, there were not observed differences between groups. However, for functional capacity (f = 0.8378, *p* < 0.001), pain (f = 4.177, *p* = 0.018), general (f = 16,876, *p* < 0.001), vitality (f = 11.164, *p* < 0.001), mental health (f = 11,209, *p* < 0.001) and total score (f = 13.441, p < 0.001), G1 differed from G2 and G3. In the domain limitation by physical aspects (f = 6.378, *p* = 0.002), G1 was significant different from G3 (Table 1).

**Table 1:** Quality of life of postmenopausal women allocated according to moderate physical activity per week (*n* = 102)

Variables	G1 (n = 34) Mean ± SD	G2(*n* = 34) Mean ± SD	G3(n = 34) Mean ± SD	F	*p*-value
**Physical activity time**					
Moderate (min/week)	72.61 ± 41.83	287.20 ± 79.31	606.56 ± 164.45	209,867	**< 0.001**^**#**^
**Quality of life**					
Functional capacity	50.47 ± 21.21^a^	61.85 ± 17.52	61.82 ± 17.15	8378	**< 0.001**
Limitations by physical aspects	53.68 ± 45.69^b^	70.59 ± 24.20	80.88 ± 18.52	6378	**0.002**
Pain	50.47 ± 21.21^a^	61.85 ± 17.52	61.82 ± 17.15	4177	**0.018**
General health status	61.56 ± 22.53^a^	81.29 ± 13.98	81.03 ± 8.41	16,876	**< 0.001**
Vitality	51.94 ± 19.03^a^	68.38 ± 18.24	71.91 ± 15.32	11,164	**< 0.001**
Social aspects	71.38 ± 22.76	77.28 ± 17.01	81.46 ± 14.43	2571	0.082
Emotional aspects	54.84 ± 45.17	64.72 ± 39.30	78.42 ± 24.48	2513	0.086
Mental health	66.24 ± 16.18^a^	76.94 ± 14.50	81.53 ± 9.38	11,209	**< 0.001**
Total score	59.75 ± 18.85^a^	72.35 ± 13.25	76.94 ± 8.35	13,441	**< 0.001**

^#^ Significant difference between groups; ^a^ Significant difference in relation to G2 and G3; ^b^ Significant difference from G3

**Fig. 2:**
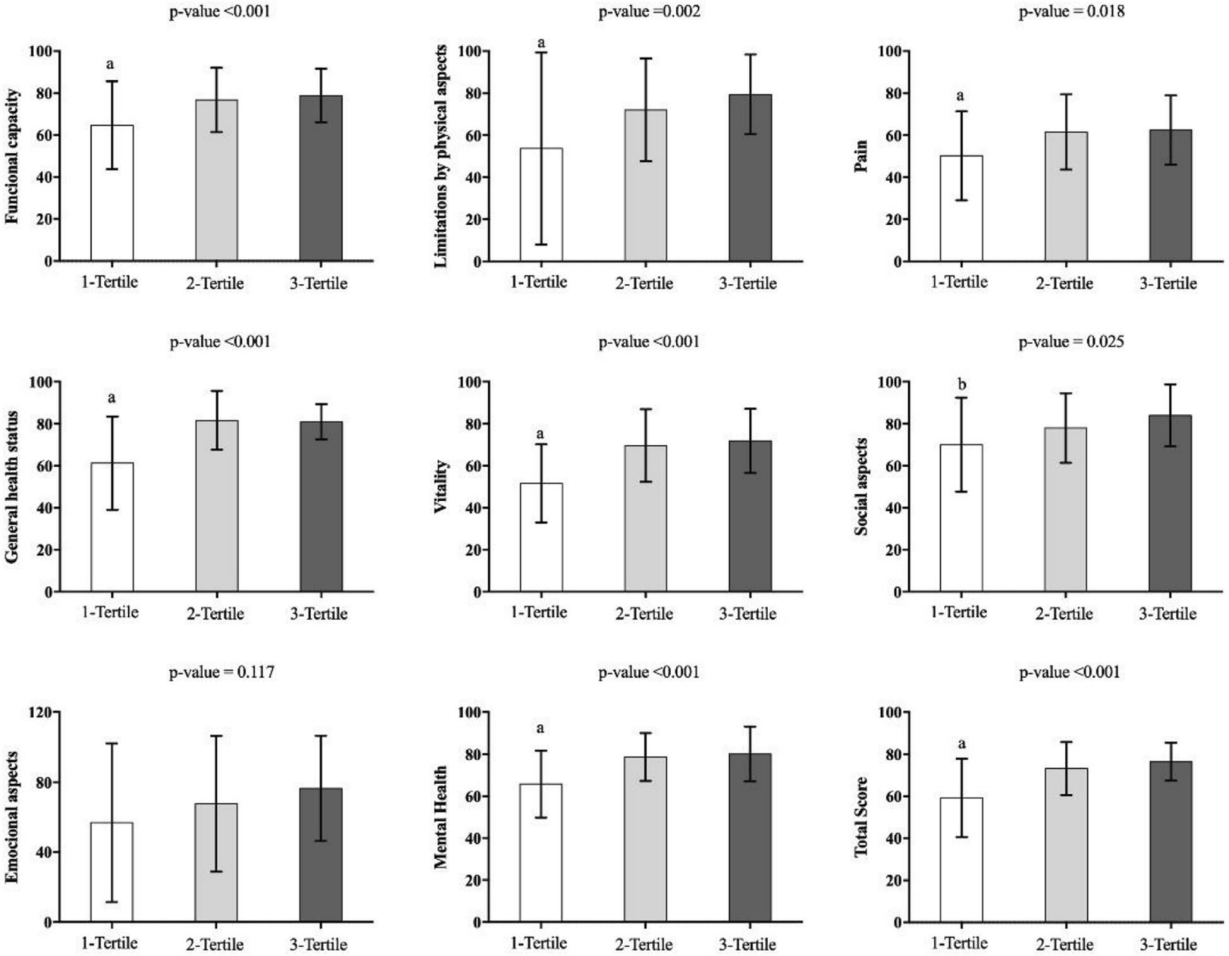
Quality of life of postmenopausal women allocated according to moderate vigorous physical activity per week (n = 102)

The MVPA of the participants presented a significant difference (f = 219.723, p < 0.001) among the three groups. In terms of QoL, only the emotional domain did not present a difference between the groups. However, for functional domains (f = 7.082, p < 0.001), limitation by physical aspects (f = 5.887, *p* = 0.004) pain (f = 4.560, *p* = 0.013), general health (f = 17.940, p < 0.001), vitality (f = 14.300, p < 0.001), mental health (f = 11.570, p < 0.001) score (f = 14.528, p < 0.001) G1 differed from G2 and G3. The social domain (f = 3.823, *p* = 0.025) showed a significant difference for G3 only (Fig. 2).

The time of mild PA, moderate and MVPA in postmenopausal women showed a positive and significant correlation for all domains (functional capacity, limitations by physical aspects, pain, general health, vitality, social aspects, emotional aspects, mental health and total score) of QoL. However, mild PA time presented more moderate correlations when compared to moderate and MVPA (Table 2).

**Table 2:** Relationship d time physical activity and quality of life of postmenopausal women (*n* = 102)

	Light		Moderate		MVPA	
Quality of life	R	*p*-value	R	*p*-value	R	*p*-value
Functional capacity	0.282	**0.004**	0,333	**< 0.001**	0.341	**< 0.001**
Limitations by physical aspects	0.272	**0.006**	0.295	**0.003**	0.298	**0.002**
Pain	0.368	**0.001**	0.235	**0.017**	0.249	**0.012**
General health status	0.585^a^	**0.001**	0.419^a^	**< 0.001**	0.448^a^	**< 0.001**
Vitality	0.604^a^	**0.001**	0.415^a^	**< 0.001**	0.448^a^	**< 0.001**
Social aspects	0.367	**0.001**	0.246	**0.013**	0.253	**0.010**
Emotional aspects	0,323	**0.001**	0.228	**0.021**	0.237	**0.016**
Mental health	0.480^a^	**0.001**	0,395	**< 0.001**	0,399	**< 0.001**
Total score	0.556^a^	**0.001**	0.438^a^	**< 0.001**	0.450^a^	**< 0.001**

^a^Moderate Correlation (FRANZBLAU, 1958) [14]

## Discussion

The aim of this study was to evaluate whether postmenopausal women who spend more time in mild, moderate, moderate+vigorous weekly physical activity have a better quality of life and associate the time in each of these activities with the domains of QoL. We found that women who spend more time in mild PA during day present higher values in all domains of QoL (functional capacity, limitations due to physical aspects, pain, general health, vitality, aspects social, emotional aspects, mental health), when compared to those that spend more time in moderate or MVPA. Women who spend more time in mild PA presented statistical difference to all domains of QoL. Furthermore, mild PA showed more number of positive significant correlations with QoL, suggesting that the effect of PA of mild intensity on the QoL of postmenopausal women is greater than the effects of moderate and MVPA.

It is known that the practice of PA and the reduction of sedentary behavior are associated with health in older adults, but it is recommended by the ACSM that people should perform a minimum of 150 min of moderate activity per week to improve health [15]. The study of Buman (2010) [16] converged with our findings as it verified that the quality of life of elderly is greater in those who remain more time in mild activity (as in leisure walks) than in moderate-vigorous activities.

Another study found that physically active women presented better scores in all domains of QoL and also verified that mild PA, although of low intensity, seems to contribute overall to better health conditions, having a positive impact in its psychic sphere and many characteristic symptoms, improving feelings and attitudes towards menopause and aging, which is reflected in the QoL [11]. In our study, the scores of social and emotional domains were grater for those that spend of more time in mild activity than in moderate and MVPA.

Mild PA may be more beneficial for the elderly, since according to Soares (2007) [17], for an elderly person practicing PA, it is not recommended to perform the exercise until exhaustion, since these activities can generate fatigue and pain, symptoms that can lead to larger complications, thus occurring the contrary to the desired effect.

The sedentary lifestyle means no enough physical activity to improve health and increase risk factors, and also when people are at rest. It is defined as the lack or decrease of PA, the individual will spend few calories per week with occupational activities [18]. Our results demonstrate that all levels of physical activity result in a better quality of life, so it is better to perform PA, regardless of intensity, than to remain sedentary behavior for a good QoL.

In a cross-sectional population-based study, Mansikkamäki et al. [19] found that women who were physically inactive were more likely to have anxiety, depression, decreased well-being, and somatic and vasomotor symptoms, results which are opposed to those of the women who were involved in the recommended amount of PA, as they have higher self-perceptive health level and better health and overall QoL. In addition, in an Australian longitudinal study on women’s health, increased PA was associated with decreased somatic symptoms [20].

This research also found that the participants of the study performed 23 min of mild activity for each minute of MVPA per week, however, there was no significant difference between the groups for body mass (data not shown). This fact may be related to the low energy expenditure of mild activities, and in order to reduce obesity, the Institute of Medicine (IOM) recommends performing 300 min of moderate to vigorous physical activity per week [21].

The practice of PA is associated to lower mortality rates in overweight or obese active individuals when compared with their sedentary pairs [22]. Thus, although longer time of mild PA does not result in a decrease in the amount of body fat, a greater active time in daily PA results in a better QoL, even if the individual remains overweight or obese [23].

The present study presents an important direction regarding the stimulus that should be given for people to remain physically active, even if it is in mild activity, as it may be more beneficial than more intense activities for postmenopausal women who are in the process of aging. In addition, another strong point of our research was the use of accelerometer, which is an objective measure that measures the amount of PA. Our results present a significant practical application that is the possibility to increase the amount of people could be in mild activity during some hours during the day, for ex. walking during working time, than wait for a leisure or rest time to exercise, that in the routine of the most the people, the time remain for it are few minutes or no time. We found that the habit to perform mild activity during the day could be better than exercise in a higher intensity for few minutes, to improve all domains of quality of life, and the importance of these finds is especially significant for aged people, for those affected by some chronic disease, mainly those that cause limitations to exercise and a compromised health-related quality of life.

Despite the importance of our results, the lack of more detailed clinical information of some variables that influence quality of life, is the main limitation. Furthermore, the study design limits interpretations due to reverse causality.

## Conclusion

In conclusion, the study showed that postmenopausal women who spend higher time of mild physical activity present better all domains of quality of life compared to moderate and moderate+vigorous intensity physical activity. The quality of life involves a complex and multidimensional panorama, and presents objective and subjective aspects. From this context, it is observed the need of studies that, besides assigning value, sustain and favor the professional actions, allowing, therefore, to understand it broadly. Also, professionals in the area should also prescribe mild activity for this population in order to improve the quality of life.

## Data Availability

Data from the current study are available upon reasonable request to the corresponding author.

## Abbreviations

PA: Physical activity
QoL: Quality of life
MVPA: Moderate vigorous physical activity
